# Epidemiological characterization of imported recurrent *Plasmodium vivax* and *Plasmodium ovale* in China, 2013–2020

**DOI:** 10.1186/s40249-021-00896-3

**Published:** 2021-08-23

**Authors:** Yanwen Cui, Li Zhang, Zhigui Xia, Hejun Zhou, Fang Huang

**Affiliations:** 1 National Institute of Parasitic Diseases, Chinese Center for Disease Control and Prevention (Chinese Center for Tropical Diseases Research), Shanghai, 200025 China; 2 NHC Key Laboratory of Parasite and Vector Biology, Shanghai, 200025 China; 3 WHO Collaborating Centre for Tropical Diseases, Shanghai, 200025 China; 4 National Center for International Research on Tropical Diseases, Shanghai, 200025 China

**Keywords:** Recurrence, Imported malaria, *Plasmodium vivax*, *Plasmodium ovale*, China

## Abstract

**Background:**

China has reached important milestones in the elimination of malaria. However, the numbers of imported recurrent cases of *Plasmodium vivax* and *P. ovale* are gradually increasing, which increases the risk of malaria re-establishment in locations where *Anopheles* mosquitoes exist. The aim of this study is to characterize the epidemiological profiles of imported recurrent *P. vivax* and *P. ovale* cases, quantifying the recurrence burden and guiding the development of appropriate public health intervention strategies.

**Methods:**

Individual-level data of imported recurrent *P. vivax* and *P. ovale* cases were collected from 2013 to 2020 in China via the Parasitic Diseases Information Reporting Management System. Demographic characteristics, temporal and spatial distributions, and the interval from previous infection to recurrence were analyzed by SAS, ArcGIS and GraphPad Prism software, respectively, to explore the epidemiological profiles of imported recurrent cases.

**Results:**

A total of 307 imported recurrent cases, including 179 *P. vivax* and 128 *P. ovale* cases, were recorded. The majority of cases occurred in males (*P. vivax* 91.1%, *P. ovale* 93.8%) and migrant workers (*P. vivax* 43.2%, *P. ovale* 44.7%). Individuals aged 30–39 years had the highest *P. vivax* and *P. ovale* recurrent infection rates, respectively. The number of imported recurrent cases of infection by these two malaria species increased from 2013 to 2018, and *P. vivax* infection showed well-defined seasonality, with two peaks in February and June, respectively. More than 90% of patients with recurrent cases did not receive radical treatment for previous infection. Most imported recurrent *P. vivax* cases were reported in Yunnan Province and were imported from Myanmar, Ethiopia, and Pakistan, while most recurrent *P. ovale* cases were reported in southern China and primarily imported from Cameroon, Ghana, and Nigeria. The intervals from previous malaria infection to recurrence among different continents were significantly different (*P* = 0.0016) for *P. vivax* malaria but not for *P. ovale* malaria (*P* = 0.2373).

**Conclusions:**

The large number of imported recurrent cases has been a major challenge in the prevention of malaria re-establishment in China. This study provides evidence to guide the development of appropriate public health intervention strategies for imported recurrent *P. vivax* and *P. ovale* cases.

**Graphic abstract:**

**Figure:**
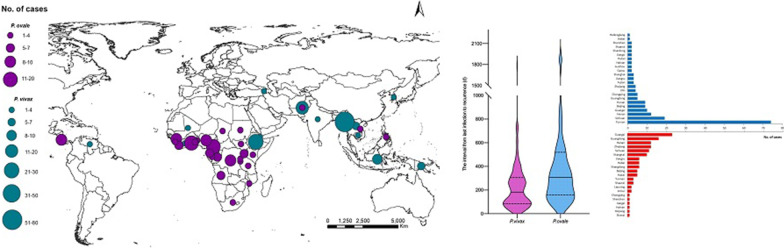

**Supplementary Information:**

The online version contains supplementary material available at 10.1186/s40249-021-00896-3.

## Background

Malaria imposes a heavy disease burden globally. In 2019, an estimated 229 million malaria cases and 409 000 malaria-related deaths were reported in 87 malaria endemic countries [1]. The majority of malaria-related morbidity and mortality is caused by two of the five *Plasmodium* species that naturally infect humans: *P. falciparum* and *P. vivax* [2]. *P. vivax* is the most geographically widespread cause of human malaria and the heaviest burden of *P. vivax* infection occurs throughout Southeast Asia, South America, and sub-Saharan Africa [3], where an estimated 2.5 billion people are at risk of infection [4].

In recent years, many countries have gradually achieved malaria pre-elimination or elimination through the upscaling of well-planned multipronged intervention strategies [5]. *P. vivax* and *P. ovale* are often the last parasites to be eliminated in malaria-elimination settings. The possibility of recurrent *P. vivax* or *P. ovale* infection in patients is a barrier to successful malaria treatment and effective control [6]. Recurrent *P. vivax* and *P. ovale* cases can be hypnozoite-derived (relapse) or caused by blood-stage treatment failure (recrudescence) or newly acquired infection (reinfection) [7]. Primaquine (PQ), the only widely available antimalarial drug with hypnozoitocidal activity for radical treatment to prevent relapse, can cause life-threatening haemolysis in humans with glucose-6-phosphate dehydrogenase (G6PD) deficiency [8]. Until now, distinguishing among these three different causes of recurrent infection has remained a challenge. Therefore, understanding the recurrence burden and epidemiological characteristics of recurrent cases will be significant in achieving malaria elimination, especially *P. vivax*.

In China, four human *Plasmodium* species (*P. vivax*, *P. falciparum*, *P. ovale* and *P. malaria*e) were endemic, and *P. vivax* was the most widely distributed. From 2001 to 2006, a local *P. vivax* outbreak in the Huang-Huai Plain in Central China occurred [9]. Since 2010, when the National Malaria Elimination Action Plan was launched, China has reached several malaria elimination milestones [10–12]. However, with increasing globalization, large numbers of individuals migrate from malaria-endemic countries or regions to China. Recurrent cases of imported *P. vivax* malaria have been reported and *P. ovale* has become the second most common of imported malaria in China, after *P. falciparum* [12]. Imported recurrent cases may increase the risks of re-establishment in malaria-free localities where *Anopheles* mosquitoes still exist. Thus, data to quantify the recurrence burden and guide the development of appropriate public health intervention strategies are urgently required [13, 14].

This study aimed to characterize the epidemiological profiles of imported recurrent *P. vivax* and *P. ovale* cases, providing evidence-based data that could support the adjustment of appropriate strategies during the post-elimination phase in China.

## Methods

### Data collection

Malaria data including *Plasmodium* species, case classification, demographic information (age, sex and occupation), radical treatment, source of malaria, date of onset, and date of previous illness from 31 provincial-level administrative divisions collected via the Parasitic Diseases Information Reporting Management System (PDIRMS) from January 1, 2013, to December 31, 2020, were reviewed. Data from Hong Kong, Macao, and Taiwan were not included in the study.

According to malaria diagnostic criteria in China [15], *P. vivax* and *P. ovale* cases were confirmed by microscopy, polymerase chain reaction (PCR), or rapid diagnostic tests (RDTs) in the laboratory. Imported malaria was defined as (i) malaria whose origin could be traced to a transmission area outside of China; (ii) a diagnosis of malaria; (iii) travel to a malaria-endemic area outside of China during the malaria transmission season; and (iv) a malaria onset time less than 1 month after returning to China during the local transmission season. In addition, according to the definition of imported cases, if the cases had a travel history to countries where malaria transmission occurs and the onset time for malaria was less than 1 month after returning to China, this country was defined as the country of origin for imported malaria cases. A recurrent case was defined as the recurrence of asexual *P. vivax* or *P. ovale* parasitaemia that was mainly caused by relapse, recrudescence or reinfection. Relapse was defined as hypnozoite recurrence. Recrudescence was defined as recurrence of asexual parasitaemia with parasites of the same genotype(s) that caused the original illness, due to incomplete clearance of asexual parasites after antimalaria treatment. Reinfection was defined as a new infection that followed the primary infection. Diagnosed *P. vivax* and *P. ovale* cases meeting any of the recurrence definitions were included in the study.

### Data analysis

The epidemiological characteristics of imported recurrent cases of *P. vivax* and *P. ovale* were analysed. Temporal distributions were analysed by year and month using the software Microsoft Excel 2010 (Microsoft, Redmond, WA, USA). Statistical analysis was performed by the statistical software SAS 9.4 (SAS Institute Inc, NC, USA). Fisher’s exact test was used to evaluate differences in demographic characteristics among the different subgroups. Interval data were visualized using the “violin plot” package in GraphPad Prism 8.4.3 (GraphPad Software, LLC., San Diego, CA, USA) to show the distribution and probability density. The Kruskal–Wallis test was used to analyse the interval distribution among different continents. Maps were created using ArcGIS 10.1 (Environmental Systems Research Institute, Inc., Redlands, CA, USA). A *P* value < 0.05 was considered to indicate a statistically significant difference.

## Results

### Demographic characteristics of imported recurrent cases

A total of 4679 imported *P. vivax* and 2202 imported *P. ovale* cases were recorded in the PDIRMS between January 1, 2013 and December 31, 2020, of which 307 were recurrent cases, including 179 recurrent *P. vivax* and 128 recurrent *P. ovale* cases. Recurrent cases accounted for 3.8% and 5.8% of the total imported cases of *P. vivax* and *P. ovale*, respectively. The majority of recurrent cases were reported in males (*P. vivax* 91.1%, *P. ovale* 93.8%). The highest numbers of recurrent *P. vivax* and *P. ovale* cases were observed in the same age group, which were patients aged 30–39 years. The proportions of patients infected with these two *Plasmodium* species in the different age groups were significantly different (*P* < 0.001). In addition, most recurrent cases occurred in workers which included migrant workers (*P. vivax* 43.2%, *P. ovale* 44.7%), construction workers (*P. vivax* 38.2%, *P. ovale* 21.3%) and other workers (*P. vivax* 19.7%, *P. ovale* 34.0%). The proportion of recurrent *P. ovale* infection was significantly different with that of *P. vivax* infection in occupation (*P* < 0.05). More than 90% of the patients with imported recurrent cases did not receive radical treatment for the previous infection (*P. vivax* 91.3%, *P. ovale* 91.7%) (Table 1).

**Table 1:** Demographic characteristics of imported recurrence cases of *Plasmodium vivax* and *P. ovale*

Demographic characteristics	Imported recurrent cases				*P* value
	*P. vivax*		*P. ovale*		
	Number	%	Number	%	
Total	179		128		
Sex					0.3869
Male	163	91.1%	120	93.8%	
Female	16	8.9%	8	6.3%	
Age (years)					< 0.0010
< 10	0	0.0%	0	0.0%	
10–19	2	1.1%	1	0.8%	
20–29	50	27.9%	21	16.4%	
30–39	51	28.5%	46	35.9%	
40–49	50	27.9%	26	20.3%	
50–59	20	11.2%	32	25.0%	
≥ 60	6	3.4%	2	1.6%	
Occupation*					< 0.0500
Migrant workers	32	43.2%	42	44.7%	
Construction workers	29	38.2%	20	21.3%	
Other workers^#^	15	19.7%	32	34.0%	
Civil servants	9	8.7%	5	4.6%	
Businessmen	5	4.9%	3	2.75%	
Others^§^	13	12.6%	7	6.4%	
Radical curative treatment*					0.9001
Yes	9	8.7%	9	8.3%	
No	94	91.3%	100	91.7%	

*Data for occupation and radical curative treatment only included data from 2017 to 2020, as they were not recorded in 2013–2016. ^#^Other workers included carpenters, electricians and miners. ^§^Others included travelers, students and migrants

### Temporal distributions of imported recurrent cases

**Fig. 1:**
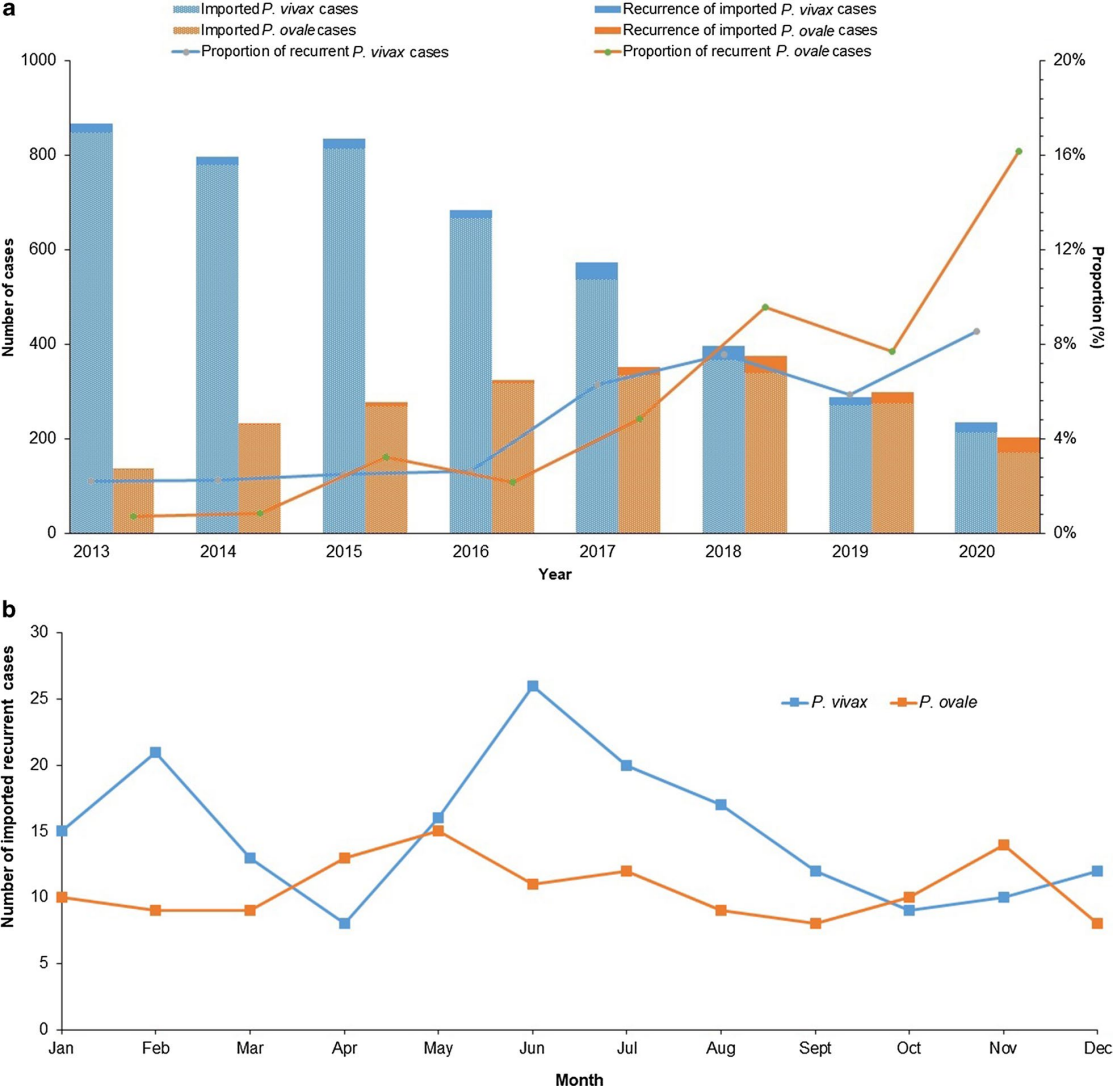
**a** Temporal distribution of imported recurrent cases from 2013 to 2020 by year. **b** Monthly distribution of imported recurrent cases from 2013 to 2020

The number of imported *P. vivax* cases showed a decreasing trend from 2013 to 2020, while the proportion of imported recurrent *P. vivax* cases increased after 2016, reaching 8.6% in 2020 (Fig. 1a). The number of imported recurrent *P. ovale* cases increased gradually from 2013 to 2018 and then slightly decreased in 2019 and 2020. This trend was mainly associated with the increasing number of imported *P. ovale* cases from 2013 to 2020. Additionally, the proportion of imported recurrent *P. ovale* cases increased continuously after 2013, reaching 16.2% in 2020.

The monthly distribution of imported recurrent cases exhibited seasonal changes from 2013 to 2020 (Fig. 1b). Recurrent *P. vivax* cases showed two peaks in February and June, accounting for 11.7% and 14.5% of the total number of imported recurrent cases of *P. vivax*, respectively. In contrast, the monthly distribution of recurrent cases of *P. ovale* over the whole year was relatively stable, with slight increases in May and November.

### Spatial distributions of imported recurrent cases

**Fig. 2:**
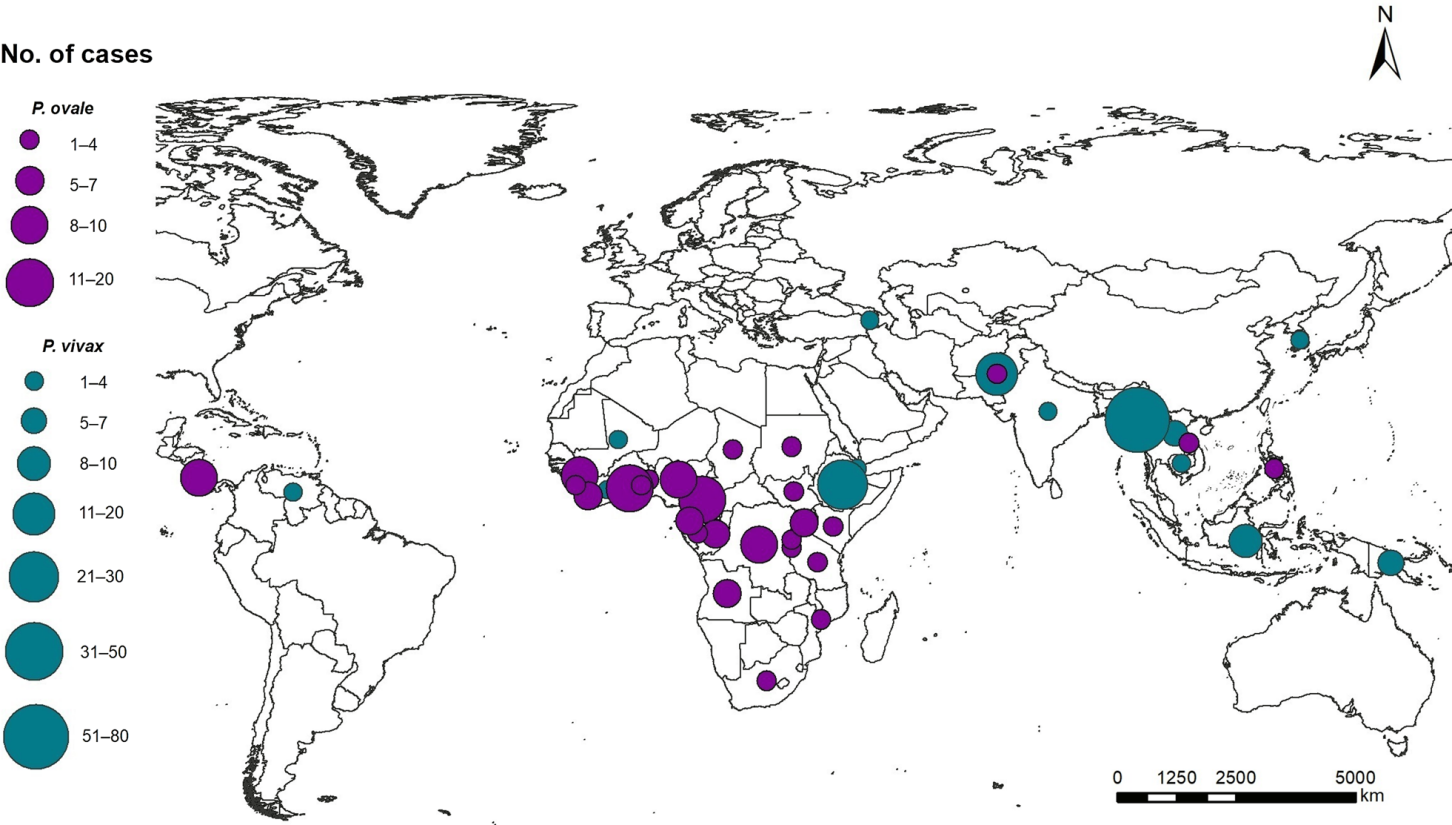
Source countries of imported recurrent *Plasmodium vivax* and *P. ovale* cases from 2013 to 2020. The world map is downloaded from https://www.naturalearthdata.com/

**Fig. 3:**
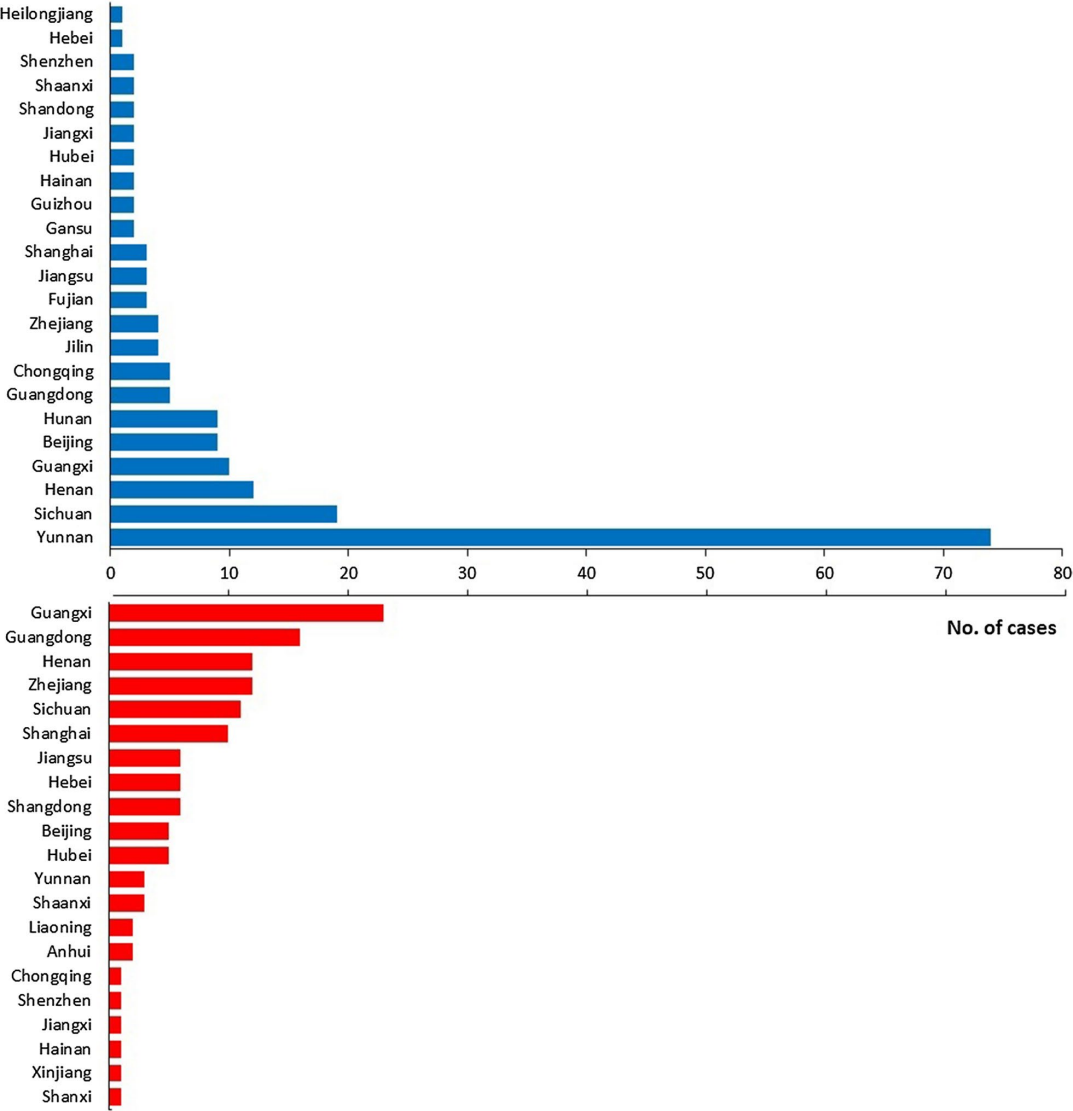
Distribution of imported recurrent *Plasmodium vivax* and *P. ovale* cases in provinces in China

The majority of recurrent *P. vivax* cases were imported from Southeast Asia (53.6%, 96/179), followed by East Africa (17.2%, 29/179), South Asia (12.4%, 21/179), West Africa (8.9%, 15/179), and Central Africa (7.1%, 12/179). Among these cases, the source countries were Myanmar, Ethiopia, and Pakistan, accounting for 43.0%, 12.9%, and 11.2%, respectively (Fig. 2 and Additional file 1). In addition, most cases were reported in Yunnan Province (41.3%), followed by Sichuan (10.6%) and Henan (6.7%) provinces (Fig. 3 and Additional file 2).

The majority of recurrent *P. ovale* cases were imported from Central Africa (40.0%, 51/128) and West Africa (39.1%, 50/128), while small proportions were from East Africa, South Africa, Asia, and North Africa, accounting for 8.9% (11/128), 8.1% (10/128), 3.2% (4/128), and 1.6% (2/128), respectively. The main source countries included Cameroon, Ghana, and Nigeria, which accounted for 13.3%, 10.9%, and 7.8%, respectively (Fig. 2 and Additional file 1). Imported recurrent cases of *P. ovale* were primarily reported in southern China, which has a large immigrant population. The main provinces included Guangxi, Guangdong, Henan and Zhejiang, accounting for 18.0%, 12.5%, 9.4% and 9.4%, respectively (Fig. 3 and Additional file 3).

### Interval from previous infection to recurrence in patients with imported recurrent cases

**Fig. 4:**
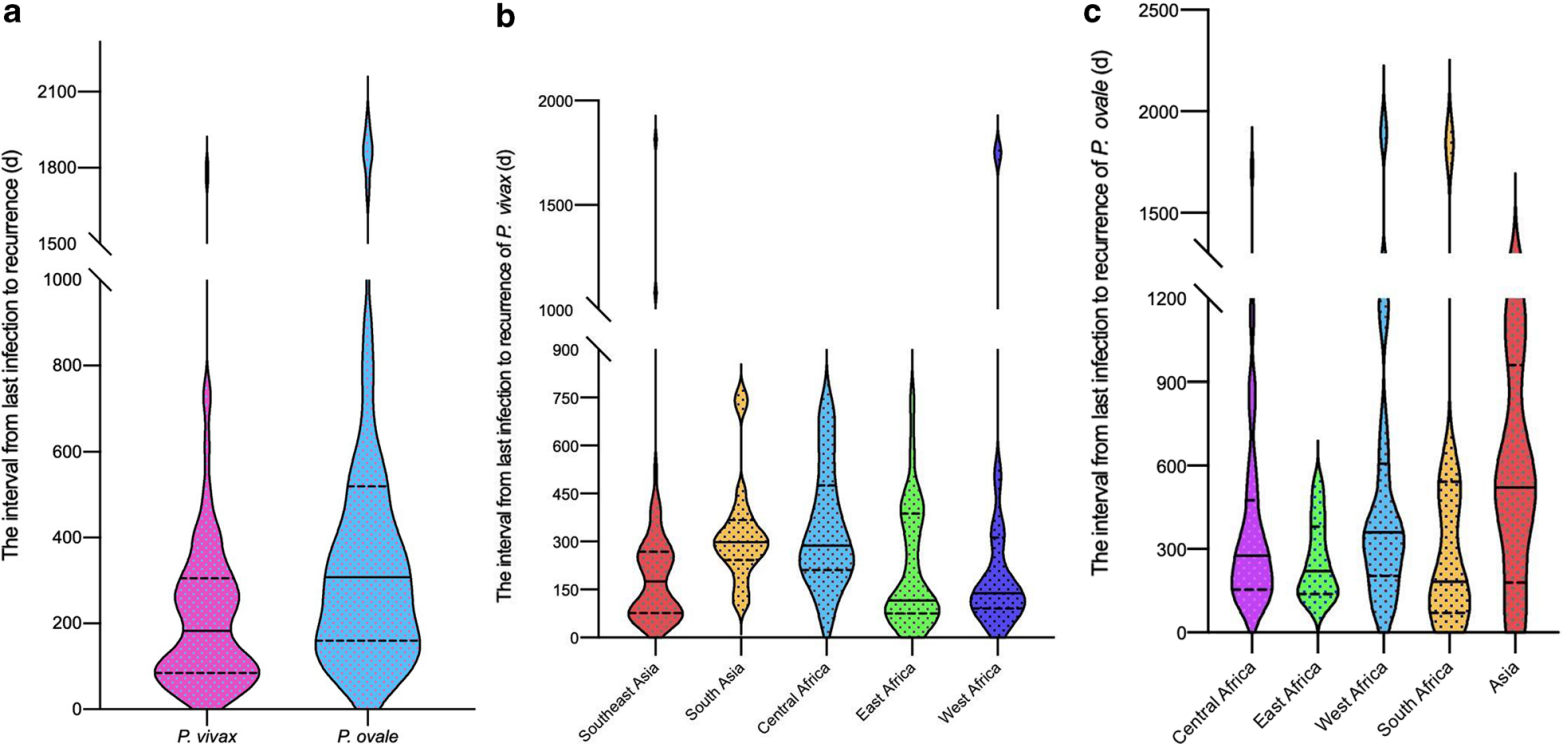
**a** Interval from previous infection to recurrence in patients with imported recurrent malaria by *Plasmodium* species. **b** Regional differences in the distributions of interval of *Plasmodium vivax* recurrence intervals. Southeast Asia includes Myanmar, Laos, Cambodia, and Indonesia. East Africa includes Ethiopia, Djibouti, Tanzania, Uganda, and Rwanda. South Asia includes Pakistan and India. West Africa includes Papua New Guinea, Sierra Leone, Liberia, Cote d’Ivoire, Ghana, Nigeria, and Mali. Central Africa includes Gabon, the Democratic Republic of the Congo, Equatorial Guinea, and Cameroon. **c** Regional differences in the distributions of *Plasmodium ovale* recurrence intervals. Central Africa includes Gabon, Democratic Republic of the Congo, Equatorial Guinea, Cameroon, and Chad. West Africa includes Guinea, Sierra Leone, Liberia, Cote d'Ivoire, Ghana, Nigeria, Benin, and Togo. East Africa includes Tanzania, Uganda, Rwanda, Burundi, and Kenya. South Africa includes Angola and Mozambique. Asia includes Pakistan, Vietnam, and the Philippines

The interval from previous infection to recurrence was recorded in only 293 patients, including 169 patients with *P. vivax* infection and 124 patients with *P. ovale* infection. The interval in *P. ovale* infection patients [308 days, interquartile range (IQR): 162–510] was longer than that in *P. vivax* infection patients (183 days, IQR: 85–305) (Fig. 4a). The *P. vivax* intervals among regions were significantly different (*P* = 0.0016), with the shortest in East Africa (116 days, IQR: 78–364), followed by West Africa (139 days, IQR: 97–248) and Southeast Asia (144 days, IQR: 75–268) (Fig. 4b). The recurrent *P. ovale* interval was shortest in South Africa (183 days, IQR: 81–521), followed by East Africa (221 days, IQR: 145–344), Central Africa (277 days, IQR: 159–475), West Africa (360 days, IQR: 218–583), and Asia (522 days, IQR: 397–679) (Fig. 4c). The median interval in the total patients with recurrent *P. ovale* infection from Africa was 308 days (IQR: 168–476). However, the interval was not significantly different among the different regions (*P* = 0.2373), and no significant difference was observed between Africa and Asia (*P* = 0.4053).

## Discussion

*P. vivax* poses a significant challenge to the elimination of malaria due to its ability to cause relapsed infection by the reactivation of dormant liver parasites called hypnozoites [16]. While China has achieved elimination, patients with *P. vivax* and *P. ovale* infections have the potential to relapse weeks, months, or years after primary infection and may not present symptoms when they arrive in China [17]. Consequently, patients and physicians are less likely to link a febrile illness with travel, resulting in delayed or missed diagnosis [18]. Therefore, it is of great significance to analyse the epidemiological characteristics of imported recurrent cases to provide evidence to guide the development of appropriate public health intervention strategies in the post-elimination settings.

In the present study, all imported recurrent *P. vivax* and *P. ovale* cases from 2013 to 2020 were included. The majority of imported recurrent cases occurred in males (*P. vivax* 91.1%, *P. ovale* 93.8%) and migrant workers (*P. vivax* 43.2%, *P. ovale* 44.7%) (Table 1), which was consistent with the distribution of total imported cases by sex (males, 94.9%) and occupation (migrant workers, 68.3%) [12]. This result may have been due to the increasing number of Chinese migrant workers that travel abroad, especially to malaria-endemic countries or regions. The groups most affected by *P. vivax* and *P. ovale* were individuals aged 30–39 years, indicating that young adults may have the highest risk of recurrent infection. One study identified that the median age of patients with imported recurrent cases in the United Kingdom from 1987 to 2015 was 32 years (IQR: 22–45) [19]. Another study found that the highest proportion of local recurrent *P. vivax* cases in Nepal occurred in individuals aged 21–30 years (35.7%), followed by 11–20 years (27%) [20]. The age group with the highest risk of recurrent malaria was similar to the age group comprising the largest proportion of the migrant population in China.

Compared with the number of recurrent cases in China before 2013 [21], the number of imported recurrent *P. vivax* and *P. ovale* cases has increased since 2013 (Fig. 1a). This rising trend might be caused by several reasons. First is the implementation of a new web-based case reporting system, PDIRMS, which has recorded information about recurrent cases since 2013. Secondly, the status of radical treatment strategies, G6PD deficiency rate, treatment adherence, and drug resistance in the infection source countries primarily associated with imported malaria cases were other key causes.

Radical treatment strategies for *P. vivax* and *P. ovale* were not the same in different countries, and even not large-scale adoption in some of the infection source countries. According to the antimalarial drug policy in China [22], recurrent *P. vivax* and *P. ovale* cases were treated with a standard regimen of 3-day chloroquine (CQ) and concurrent 8-day PQ (22.5 mg/day) for radical cure, while the first-line treatment for *P. vivax* malaria in most countries, including Myanmar, the main source country of recurrent *P. vivax* infections, is 3-day CQ and 14-day PQ (3.5 mg/kg total dose) [23, 24]. Most recurrent *P. ovale* cases were imported from Central Africa (Cameroon) and West Africa (Ghana, Nigeria) (Fig. 2). However, PQ is not used for radical cure of *P. vivax* and *P. ovale* malaria in Cameroon, Ghana, and Nigeria [25]. This may explain the increasing number of imported recurrent cases from these countries, which have a low rate of radical cure (*P. vivax* 8.7%, *P. ovale* 8.3%).

G6PD deficiency greatly hinders the widespread use of PQ, because PQ could cause haemolysis in people with G6PD deficiency [8]. The prevalence of G6PD deficiency was relatively high in the major source countries [26], including Myanmar (3–7%), Cameroon (10–13%), Ghana (17–20%), and Nigeria (13–17%). Moreover, a high prevalence of G6PD deficiency (29.6%) was detected in the Kachin ethnic group (Jingpo) along the China-Myanmar border [27]. Thus, radical cure cannot be widely adopted and patients are reluctant to take PQ, especially if they cannot be tested for G6PD deficiency [28]. In accordance with the malaria elimination programme in Myanmar, *P. vivax* patients were given a weekly dose of 0.75 mg/kg PQ for 8 weeks by village health volunteers or integrated community malaria volunteers in the community; this regimen may be widely used and safer in those without G6PD deficiency testing [29].

In addition, PQ is generally prescribed as a 14-day course for radical treatment, which hampers treatment adherence because most patients discontinue their medication as soon as their symptoms disappear. In China, an 8-day course of PQ was used to improve treatment adherence [22], and one study also showed that a short 7- to 9-day short course of PQ with CQ was equally as effective as the 14-day regimen in preventing relapse [30].

Drug resistance causes recrudescence (blood-stage treatment failure), another kind of recurrence. CQ resistance was first reported in Papua New Guinea in 1989 [31]. It then spread to Indonesia, Brazil, Myanmar, India, Cambodia, and Ethiopia [32–37]. Although imported recurrent *P. vivax* malaria in China was mainly imported from Myanmar, *P. vivax* was mostly sensitive to CQ, with treatment failure rates of less than 5% along the China-Myanmar border [38] and 2.6% in northeast Myanmar [39]. Although the CQ/PQ treatment failure rate in patients with *P. vivax* infection has been relatively low near the China–Myanmar border, CQ-resistant *P. vivax* has emerged in Greater Mekong subregion (GMS) and some countries have reported a highly resistant phenotype. In this study, most recurrent *P. ovale* cases were primarily imported from Cameroon, Ghana, and Nigeria, while there have been few reports about the CQ resistance profile of *P. vivax* and *P. ovale* in Cameroon, Ghana, and Nigeria, as *P. falciparum* is the predominant species in such areas. In addition, artemisinin combination therapies (ACTs) were recommended by WHO as the first- and second-line treatment for uncomplicated *P. falciparum* malaria as well as for chloroquine-resistant *P. vivax* malaria, which have already been used to treat infections by *P. falciparum* and mixed species resistant to CQ in Cameroon [40].

This study found that the number of imported recurrent cases of *P. vivax* displayed well-defined seasonality, with two peaks in February and June, respectively (Fig. 1b). The number of recurrent *P. ovale* cases increased slightly in May and November; these increases were related to holidays associated with family visits, increasing the number of imported cases. According to the analysis of imported malaria cases in China, imported malaria cases associated with the end of the work season or studying abroad were concentrated in May–July, while malaria cases associated with the Spring Festival/summer vacation or a return home to visit relatives were mostly distributed in January and May [41].

The intervals between infections in those with imported recurrent *P. vivax* cases among different continents were significantly different and may have been influenced by geographic and environmental factors [42–44]. Previous studies found that temperate and subtropical strains of *P. vivax* often exhibited either a long incubation or latent period of around eight to ten months, while tropical strains were characterized by short incubation times and short latency (approximately three to six weeks). In addition, Recurrence frequency may result from evolved responses to average transmission season duration and/or vector suitability periods. The median time from primary infection to relapse in patients with *P. vivax* malaria was different among nine different regions, i.e., 107 days and 41 days in sub-Saharan Africa and Southeast Asia, respectively [45]. In the present study, the median infection interval in Southeast Asia was 144 days, which was relatively long, possibly because the data analysed herein comprised the interval from previous infection to recurrence instead of from primary infection to relapse, and the data were collected from patient’ self-reports rather than a cohort study.

Some limitations were noted in this study. First, genotyping methods have not been able to precisely differentiate relapse from new infection or recrudescence. None of the imported recurrent cases in this study were stratified into relapse, recrudescence, or reinfection subgroups. Secondly, information for tracing the infection source countries of imported malaria cases is not available. Thirdly, data on the interval from previous infection to recurrence were collected based on patient self-reports rather than a cohort study. In addition, *P. ovale curtisis* and *P. ovale wallikeri*, two genetically distinct subspecies of *P. ovale*, were not differentiated in the study.

## Conclusions

Large numbers of imported recurrent cases have been a major challenge in achieving and maintaining malaria elimination in China. This study provides data to guide the development of appropriate public health intervention strategies for imported recurrent *P. vivax* and *P. ovale* cases. A surveillance system based on the “1-3-7” approach should be fully operational with integrated drug efficiency surveillance to identify recurrent cases in the post-elimination phase. In addition, the radical cure rate and the capability of G6PD deficiency testing in patients with imported cases should be strengthened. Targeted intervention strategies combined with powerful surveillance could sustain malaria elimination and prevent re-establishment in China.

## Supplementary Information

**Additional file 1.** Distribution of imported recurrent *Plasmodium vivax* cases at the provincial level in China.

**Additional file 2.** Distribution of imported recurrent* Plasmodium ovale* cases at the provincial level in China.

**Additional file 3.** Numbers of imported recurrent* Plasmodium vivax* and* P. ovale* cases from different source countries from 2013 to 2020.

## Data Availability

The datasets used and/or analysed during the current study are available from the corresponding author upon reasonable request.
