# Supplementary material for: Epidemiological characterization of imported recurrent Plasmodium vivax and Plasmodium ovale in China, 2013–2020
Source: Infect Dis Poverty. 2021 Aug 23;10:113. doi: 10.1186/s40249-021-00896-3 (PMC8381563; doi:10.1186/s40249-021-00896-3)
Supplement: Supplementary file 1 — Additional file 1. Distribution of imported recurrent Plasmodium vivax cases at the provincial level in China. [file 40249_2021_896_MOESM1_ESM.docx]

**Additional file 3: The number of imported recurrent *P. vivax* and *P. ovale* cases in different source countries from 2013 to 2020**

| **Source countries** | **Number of recurrent *P. vivax* cases** | **Number of recurrent *P. ovale* cases** |
| --- | --- | --- |
| Angola | 1 | 5 |
| Armenia | 1 |  |
| Benin | 0 | 1 |
| Burundi | 0 | 1 |
| Cambodia | 4 | 0 |
| Cameroon | 2 | 17 |
| Central Africa | 0 | 2 |
| Chad | 0 | 3 |
| Congo | 2 | 7 |
| Costa Rica | 0 | 8 |
| Côte d'Ivoire | 1 | 0 |
| Dem. Rep. Congo | 4 | 9 |
| Djibouti | 1 | 0 |
| Eq. Guinea | 3 | 7 |
| Ethiopia | 23 | 0 |
| Gabon | 1 | 4 |
| Ghana | 1 | 14 |
| Guinea | 0 | 9 |
| India | 1 | 0 |
| Indonesia | 10 | 0 |
| Kenya | 0 | 1 |
| Korea | 1 | 0 |
| Lao PDR | 6 | 0 |
| Liberia | 1 | 5 |
| Mali | 1 | 0 |
| Mozambique | 0 | 4 |
| Myanmar | 77 | 0 |
| Nigeria | 2 | 10 |
| Pakistan | 20 | 2 |
| Papua New Guinea | 7 | 0 |
| Philippines | 0 | 1 |
| Rwanda | 1 | 1 |
| Sierra Leone | 2 | 4 |
| Solomon Is. | 1 | 0 |
| S. Sudan | 0 | 1 |
| South Africa | 0 | 1 |
| Sudan | 1 | 1 |
| Tanzania | 1 | 2 |
| Togo | 0 | 1 |
| Uganda | 2 | 6 |
| Venezuela | 1 | 0 |
| Vietnam | 0 | 1 |
| Total | 179 | 128 |
