# Supplementary figures and images for: Epidemiological characterization of imported recurrent Plasmodium vivax and Plasmodium ovale in China, 2013–2020
Source: Infect Dis Poverty. 2021 Aug 23;10:113. doi: 10.1186/s40249-021-00896-3 (PMC8381563; doi:10.1186/s40249-021-00896-3)

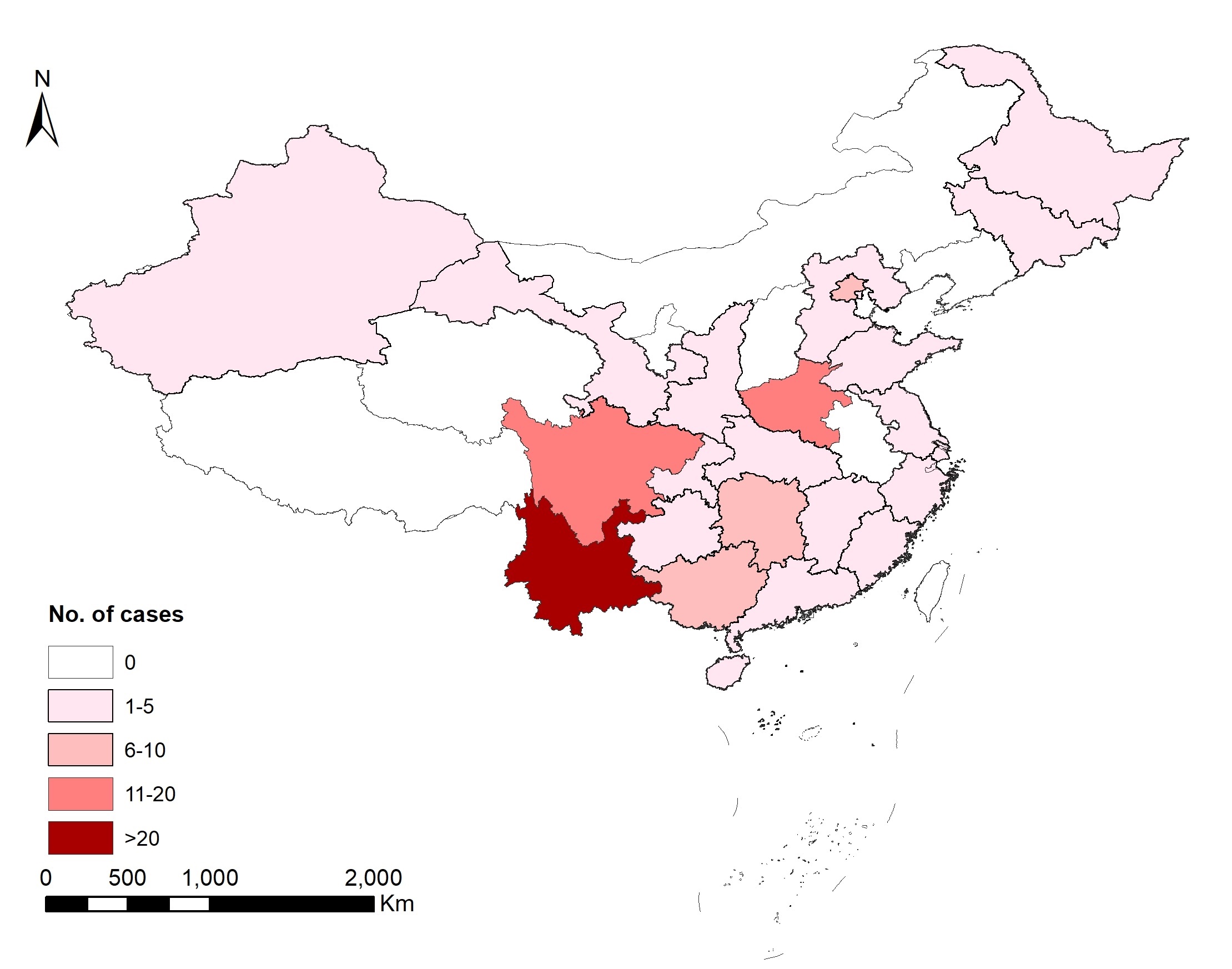

Supplement: Supplementary file 2 — Additional file 2. Distribution of imported recurrent Plasmodium ovale cases at the provincial level in China. [file 40249_2021_896_MOESM2_ESM.jpg]

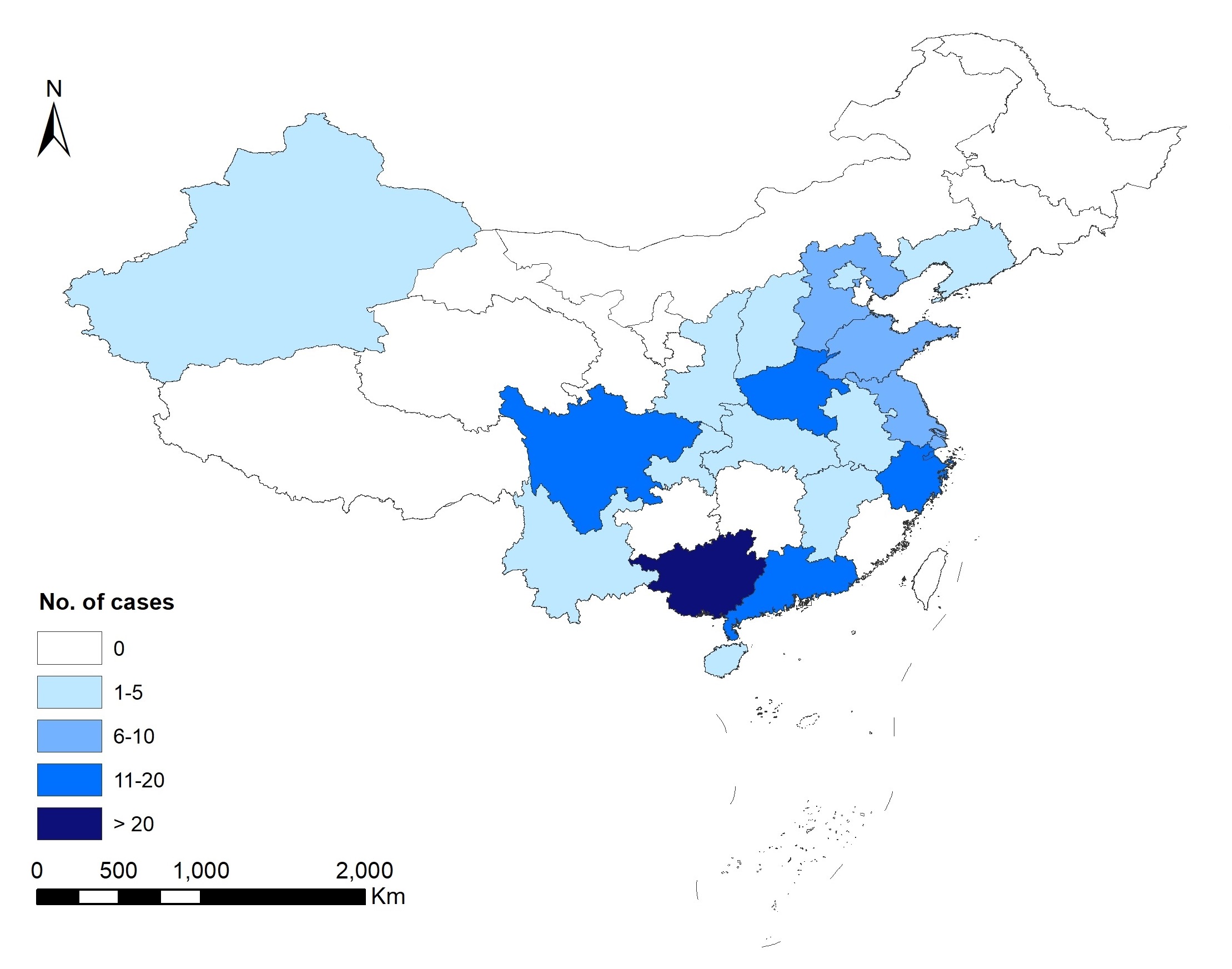

Supplement: Supplementary file 3 — Additional file 3. Numbers of imported recurrent Plasmodium vivax and P. ovale cases from different source countries from 2013 to 2020. [file 40249_2021_896_MOESM3_ESM.jpg]
